# First person – Francesco Chiani and Tiziana Orsini

**DOI:** 10.1242/dmm.041442

**Published:** 2019-08-02

## Abstract

First Person is a series of interviews with the first authors of a selection of papers published in Disease Models & Mechanisms (DMM), helping early-career researchers promote themselves alongside their papers. Francesco Chiani and Tiziana Orsini are co-first authors on ‘Functional loss of *Ccdc151* leads to hydrocephalus in a mouse model of primary ciliary dyskinesia’, published in DMM. Francesco is a researcher (PhD) in the lab of Fabio Mammano (Italian IMPC/INFRANFRONTIER delegate) at the Institute of Biochemistry and Cell Biology – Italian National Research Council, Monterotondo, Rome, Italy, where he is interested in engineering and studying novel mouse models for human disease with the aim to understand gene function in normal physiology and pathological conditions. Tiziana is a CNR Technologist in the lab of Fabio Mammano, studying the function of genes in normal conditions and during disease development in mouse models by applying the micro-computed tomography (micro-CT) imaging technique.


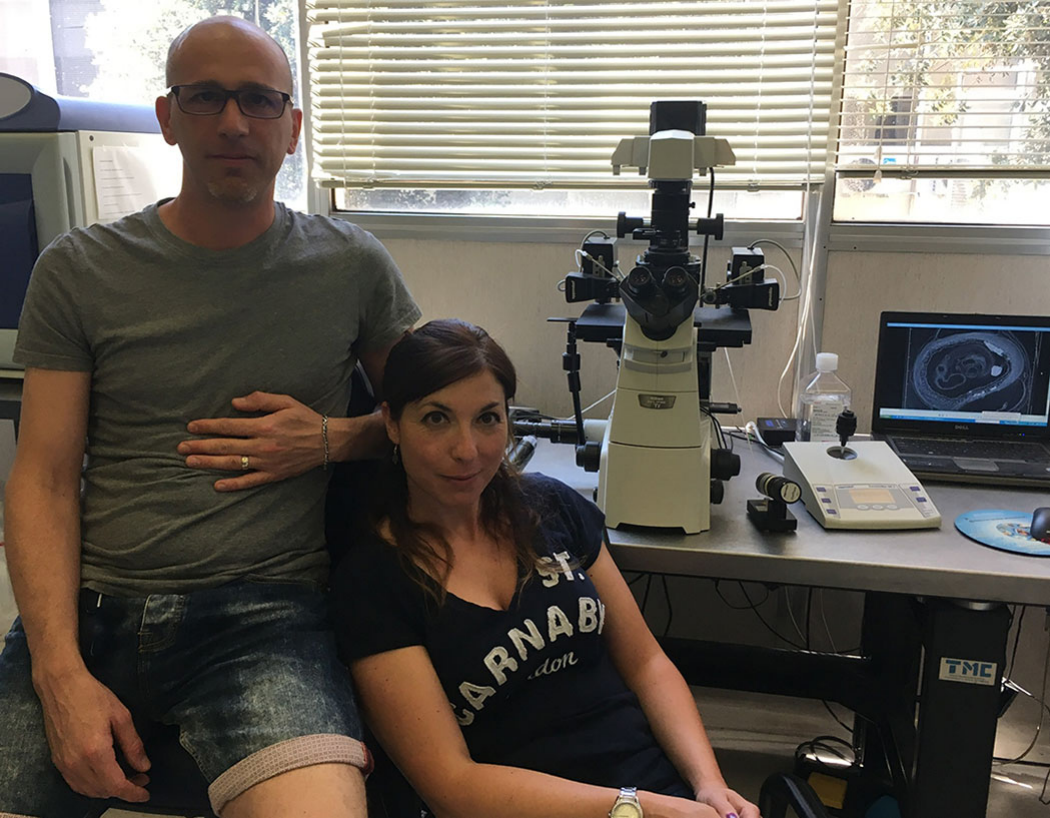


**Francesco Chiani and Tiziana Orsini**

**How would you explain the main findings of your paper to non-scientific family and friends?**

F.C. & T.O: Mutation in the *Ccdc151* gene leads to primary ciliary dyskinesia (PCD) in patients. PCD is a complex genetic disease that originates from defects in motile cilia, responsible for fluid flow along the epithelial surfaces in many organs and systems. In the nervous system, cilia are essential for the normal flow of cerebrospinal fluid (CSF) within the ventricular brain system into the central canal of the spinal cord, while in lungs and ears cilia are required for mucus and fluid removal from these organs. In the female and male reproductive systems, migration of the egg and sperm require normal function of the cilia.

To understand the function of the *Ccdc151* gene, we have engineered a mouse model in which it is deleted. This mouse model recapitulates many features of the PCD, which are often observed in patients with a mutation in the *Ccdc151* gene. Animals with the deletion of the gene developed a left-right body asymmetry defect and lethal congenital hydrocephalus. To study the structure of the normal and hydrocephalic brain in three-dimensional mode, we applied X-ray micro-CT, which facilitates analyses of hydrocephalic brain morphology.

Another advantage of the presented animal model is also in the possibility to conditionally remove the *Ccdc151* gene in adult animals to overcome the early lethality observed in *Ccdc151*-knockout animals. Conditional deletion of the *Ccdc151* gene in adult males leads to defects in spermatogenesis, with fewer sperm cells and low sperm motility. Overall, the presented animal model allows study of the role of the *Ccdc151* gene in the development of PCD disease during development and in adult animals.

“This animal model will be useful for studying mechanisms underlying hydrocephalus, a condition whose treatment has not changed for decades.”

**What are the potential implications of these results for your field of research?**

F.C. & T.C: The results presented here demonstrate the role of the *Ccdc151* gene in the development of different phenotypic features of PCD disease. Especially striking is the progression of the congenital hydrocephalus in the mutant animals. This animal model will be useful for studying mechanisms underlying hydrocephalus, a condition whose treatment has not changed for decades. In this work we also presented a novel method for studying the expression pattern of the *Ccdc151* gene using X-ray micro-CT directly in 3D in intact murine brain *ex vivo*. This method is based on generating a molecular signal within the mouse brain through the reaction catalyzed by the bacterial enzyme β-galactosidase (encoded by *lacZ*). In this animal model, knockout of the *Ccdc151* gene is achieved by replacement of the two critical exons of *Ccdc151* by the *lacZ* gene, therefore resulting in a *Ccdc151-lacZ* reporter allele. Products of the β-galactosidase reaction are detected as high-density areas detectable by X-ray microCT imaging. This methodology has allowed us to analyze the expression of the *Ccdc151* gene in whole mouse brain. This microCT imaging methodology could be applied to facilitate studies on gene expression directly in the intact brain carrying the *lacZ* reporter gene, which is widely used as a reporter gene in mouse models.

“This mouse model could also be instrumental to address whether species-specific genetics or anatomical differences might explain why human PCD does not occur with severe hydrocephalus, as is often observed in mice.”

**What are the main advantages and drawbacks of the model system you have used as it relates to the disease you are investigating?**

F.C. & T.O.: The *Ccdc151*-knockout mouse model faithfully recapitulates several features of human PCD disease. The availability of this animal model will allow researchers to further dissect the mechanisms by which pathological conditions develop in different organs. Since the *Ccdc151^−/−^* animals die early in life due to severe hydrocephalus, the conditional *Ccdc151*-knockout animals described in our work are an especially valuable tool to study the possible consequences of the ciliopathies in adulthood. We are also aware of the fact that, while in mice we have observed severe hydrocephalus, this defect was not observed in patients with the *Ccdc151* gene mutation. Moreover, in humans, the genes involved in the mechanics of ciliary movements is rarely associated with the hydrocephalus disease. Therefore, to what extent defective ependymal ciliary motility contributes to the development of congenital hydrocephalus is still a matter of debate. This mouse model could also be instrumental to address whether species-specific genetics or anatomical differences might explain why human PCD does not occur with severe hydrocephalus, as is often observed in mice.

**What has surprised you the most while conducting your research?**

F.C.: The severe hydrocephalus observed upon *Ccdc151* gene deletion was rather unexpected. Another group, who studied the mouse model with a loss-of-function point mutation in the *Ccdc151* gene (*Ccdc151^Snbl^* mouse), did not observe the hydrocephalus phenotype. The reason for this can be explained by the differences in genetic background of the two *Ccdc151* loss-of-function models. Further studies will allow us to dissect the genes that might cooperate with *Ccdc151* in the brain that are important for hydrocephalus development.

T.O.: It was also astonishing to observe that *Ccdc151-lacZ* gene expression can be detected by X-ray imaging. By analyzing microCT images, we observed an increase of the X-ray detected densities in the same regions where we have seen expression of the *Ccdc151-lacZ* reporter gene in histological analysis. This is how we came to the conclusion that *lacZ* reporter gene expression can be performed directly in intact mouse brain using microCT *ex vivo*.

“Application of microCT technology greatly facilitated these studies because it allows us to collect volumetric information in developing brains, providing morphological and quantitative data simultaneously.”

**Describe what you think is the most significant challenge impacting your research at this time and how will this be addressed over the next 10 years?**

F.C.: While it is now clear that loss of *Ccdc151* gene function leads to defective ciliary motility, little is known about the mechanism by which *Ccdc151* contributes to the formation of functional cilia. It is now known that *Ccdc151* is necessary for outer dynein arm formation, but the precise mechanism by which Ccdc151 protein accomplishes its function is not known and will be addressed in future research. In addition, the conditional *Ccdc151*-knockout mouse model can be instrumental to dissect the role of the motile cilia in diverse physiological processes during development, adult life and aging.

T.O.: I will continue to study the morphological consequences of the loss of the *Ccdc151* gene in different organs using microCT imaging. We aimed to continue the studies using the *Ccdc151*-knockout mouse model and microCT imaging to understand hydrocephalus onset during development. Application of microCT technology greatly facilitated these studies because it allows us to collect volumetric information in developing brains, providing morphological and quantitative data simultaneously.

**Figure:**
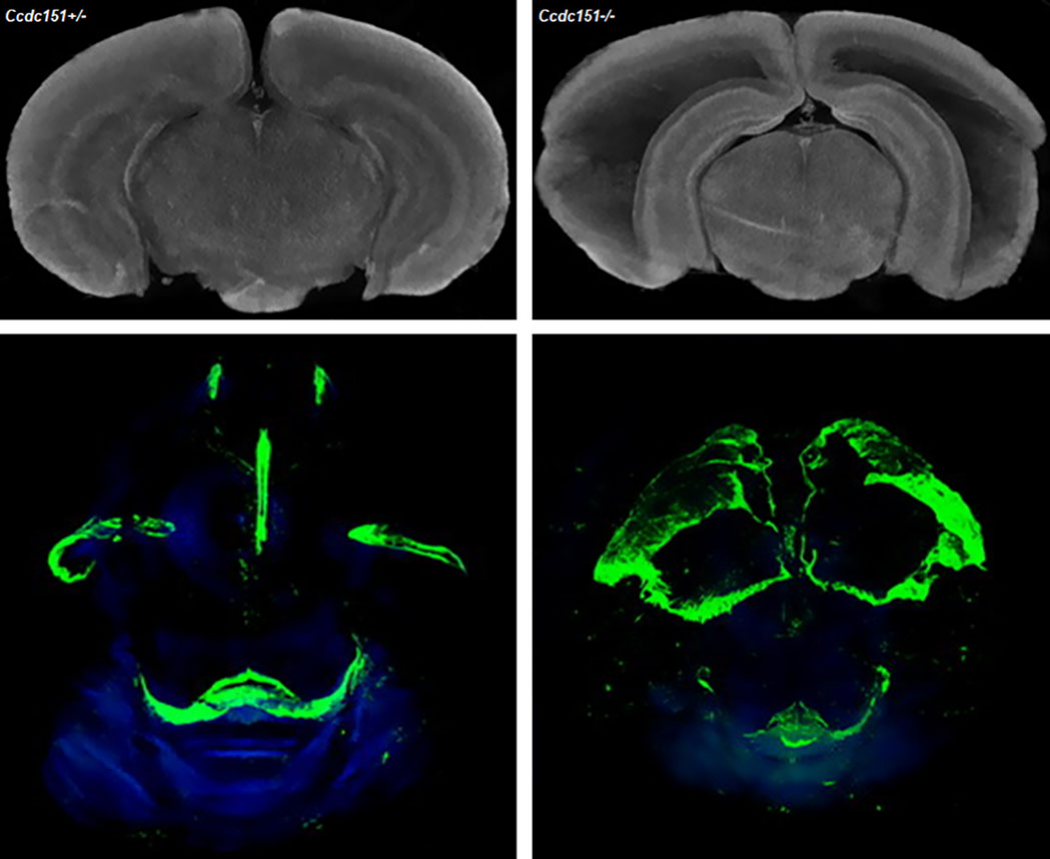
**Three-dimensional microCT imaging of *Ccdc151*-knockout mouse brain detects lateral ventricle enlargement, indicating severe hydrocephalus. MicroCT imaging detects *Ccdc151-lacZ* gene expression in cells lining the ventricular brain system.**

**What changes do you think could improve the professional lives of early-career scientists?**

F.C.: I am currently a member of the worldwide International Mouse Phenotyping Consortium (IMPC) and am actively involved in the process of engineering and producing disease mouse models. I think that my career could benefit greatly from the possibility of open communication with other scientists by attending scientific meetings and conferences, and by expansion of large international projects for the broader scientific community.

T.O.: I think it is essential to invest in the formation of a strong professional experience for early-career researchers, thus increasing the diversification of laboratory life at the academic level. I also feel that the creation of the scientific networks for early-career researchers to discuss the challenges and opportunities for career advances will probably be beneficial for young scientists at the beginning of their careers.

**What's next for you?**

F.C.: I will continue to produce and share the tools, such as mouse models, and disseminate these tools within the broad scientific community. In addition, I would like to implement the analysis of the data already produced by the scientific community with the novel big data analysis methodology, such as artificial intelligence approaches. Recently, a large amount of data became available to the scientist worldwide that required development of novel methods of large-dataset analysis and data interpretation, which will allow for understanding the processes and the patterns hidden in the often unordered collections of data.

T.O.: I just finish my postdoctoral training and accepted a position as a permanent member of the largest Italian public multidisciplinary research network, Italian National Research Council (CNR). Being a permanent member of CNR gives me great possibilities as an early-career researcher to learn from the established members and to build upon the knowledge accumulated among CNR scientists. I am aiming to develop novel X-ray microCT-based methodology to study gene expression in intact organs in mouse models. This is an important challenge that I will be facing over the next few years. The CNR is a perfect place to achieve these aims since its multidisciplinary nature will greatly facilitate my goals.
